# Severe Delayed Hemolysis Associated with Regulated Parenteral Antimalarial Drug

**DOI:** 10.3201/eid2101.140147

**Published:** 2015-01

**Authors:** Julia Lee, Sigmund Krajden, Christopher Graham, Andrea K. Boggild, Katerina Pavenski, Jay S. Keystone, Kevin C. Kain

**Affiliations:** University of Toronto, Toronto, Ontario, Canada (J. Lee, A.K. Boggild, J.S. Keystone, K.C. Kain);; McMaster University, Hamilton, Ontario (J. Lee);; St. Joseph's Health Centre, Toronto (S. Krajden);; Trillium Health Partners, Mississauga, Ontario, Canada (C. Graham);; Public Health Ontario, Toronto (A.K. Boggild);; St. Michael’s Hospital, Toronto (K. Pavenski);; University Health Network-Toronto General Hospital, Toronto (A.K. Boggild, J.S. Keystone, K.C. Kain)

**Keywords:** malaria, artesunate, artemisinin, antimalarial, hemolysis, hemolytic anemia, Good Manufacturing Practice, GMP, *Plasmodium falciparum*, vectorborne, mosquito, parasite, protozoan

**To the Editor:** Intravenous artesunate is recommended by the World Health Organization as first-line treatment for severe malaria. While artemisinin-based therapies are well tolerated, recent studies have reported cases of severe delayed hemolysis after artesunate treatment for malaria (*1*–*5*). To date, all reported cases have been associated with the use of artesunate not produced under Good Manufacturing Practice (GMP) standards. The United States and Canada are the only countries that use GMP artesunate, and a recent review concluded that delayed hemolysis may be related to differences in GMP versus non-GMP artemisinins (*5*). We report a case of severe delayed hemolysis after administration of GMP artesunate to treat a patient with severe malaria.

During October 2012, A previously healthy, 31-year-old Canadian-born man sought treatment at a hospital in Toronto, Ontario, Canada, after 3 days of fever and severe headaches. He had returned 10 days earlier from a 10-day work trip to South Sudan. He did not use malaria chemoprophylaxis while there but did sleep under an insecticide-treated net. During initial assessment, a blood smear showed *Plasmodium falciparum* malaria with parasitemia of 22% (1,100,000 parasites/μL) and the following levels: bilirubin, 88 (reference range 5–17) μmol/L; aminotransferase, 105 (reference range 10–38) U/L; creatinine, 130 (reference range 80–115) μmol/L; hemoglobin, 144 (reference range 140–160) g/L, and a platelet count of 17 × 10^9^/L (reference range 150–450 × 10^9^/L). In the emergency department, he was given 1 dose each of doxycycline, atovaquone/proguanil, and artemether/lumefantrine; within an hour of ingestion of these drugs, he vomited. He was transferred to a tertiary level hospital for admission to the intensive care unit and exchange transfusion. Intravenous artesunate was administered (2.4 mg/kg at 0, 12, 24, and 48 h), then a 3-day course of oral atovaquone/proguanil was ordered. On admission, his chest radiograph showed no abnormalities, and blood cultures were negative; his hemoglobin level was 125 g/L; no treatment was initiated for decreased hemoglobin. Parasitemia was undetectable within 36 hours of admission to the intensive care unit. The patient was discharged 5 days later.

Four days after discharge, the patient returned to the tertiary level hospital seeking treatment. He reported that beginning 2 days after discharge, he had fever and “merlot-colored” urine. On admission, he was noted to be jaundiced. Laboratory values included levels of bilirubin of 89 μmol/L, lactate dehydrogenase (LDH) of 1,976 (reference 120–240) U/L, hemoglobin of 81 g/L and marked hemoglobinuria. Multiple thick and thin blood smears were negative for *Plasmodium* spp. 

During the course of his second admission, he required 8 blood transfusions to maintain his hemoglobin level above 75 g/L. He continued to have unexplained hemolysis and hemoglobinuria: laboratory results showed a nadir of hemoglobin at 68 g/L and an LDH peak of 3,429 U/L and a low haptoglobin level (<0.12 g/L [reference 0.3–2.0 g/L]). His glucose-6-phosphate dehydrogenase level was within reference range. Supportive therapy was continued, and hemolysis ceased spontaneously 10 days after onset. When seen during a follow-up visit 6 weeks later, he was asymptomatic and his hemoglobin level was 135 g/L. Pre- and post-transfusion and follow-up testing did not show evidence of red blood cell alloantibodies, making the possibility of a delayed hemolytic transfusion reaction unlikely. Serologic tests showed that the he was also positive for causative organisms for schistosomiasis, strongyloidiasis, and Q-fever. These diagnoses were consistent with past infections and were not considered to be contributory to the current severe hemolytic event.

In all previous case reports of delayed hemolysis, patients received World Health Organization-prequalified, but not GMP-certified, artesunate (*1*–*5*). In this report, the parenteral drug used was GMP certified and produced by the US Army Medical Materiel Development Activity. A diagnosis of artesunate-associated hemolysis was made in this case based on the temporal relationship with therapy and the absence of other identified causes of intravascular hemolysis. His time course of hemolysis after treatment corresponds with recent case series in Europe (*1*–*4*): his hemoglobin level reached a nadir at approximately day 15. The outcome of this case corresponds with a proposed case definition by Rolling and colleagues to distinguish artesunate-related hemolysis from that attributable to malaria infection alone (*4*). 

We suggest a case definition whereby a decrease in hemoglobin combined with an increase in LDH between week 2 and 3 is characteristic of delayed hemolysis associated with artesunate. Because treatment for severe malaria is not given as monotherapy, we cannot exclude a potential contributory role of the other antimalarial agents he received. However, severe intravascular hemolysis has rarely been reported in relationship to these agents. Additionally, we cannot exclude a potential role for drug-induced immune hemolysis. Nonetheless, given the severity of the hemolysis and the delayed onset, health care workers should be cognizant of this late, potentially life-threatening complication of artemisinin-based therapy. All patients treated with artesunate for severe malaria should be monitored for 4 weeks and evaluated for hemolytic anemia.
